# The Mechanism of the Rappe Rearrangement—A Stereochemical Investigation Using Density Functional Theory

**DOI:** 10.1021/acs.joc.5c00726

**Published:** 2025-06-20

**Authors:** Toby J. Sommer, Henry S. Rzepa

**Affiliations:** † Independent Researcher, P.O. Box 541092, Waltham, Massachusetts 02454, United States; ‡ Department of Chemistry, Imperial College, Molecular Sciences Research Hub, White City Campus, 82 Wood Lane, London W12 0BZ, England

## Abstract

Since it was first described in the 1950s, there has been no explanation of the extraordinarily high selectivity for the formation of the thermodynamically less stable *cis*-2-alkenoic acids from the treatment of α,α′-dibromoketones with aqueous base. Very early on, it was suggested that such specificity must arise via “concerted processes” but without elaboration. Provided here is a detailed mechanistic description of the reaction based on computational insight, identifying a stereospecific first step for the ring closure to a 3-ring cyclopropanone, which follows Woodward–Hoffmann rules for a two-electron allylic cation, accompanied by departure of a bromine atom in an S_N_2-like process. The stereochemistry established in this step persists in the second step, which involves ring opening of the 3-ring to give a *cis*-2-alkenoic acid. Other mechanistic pathways have higher energies. With this new understanding, further synthetic applications of the Rappe Rearrangement are suggested.

## Graphical abstract

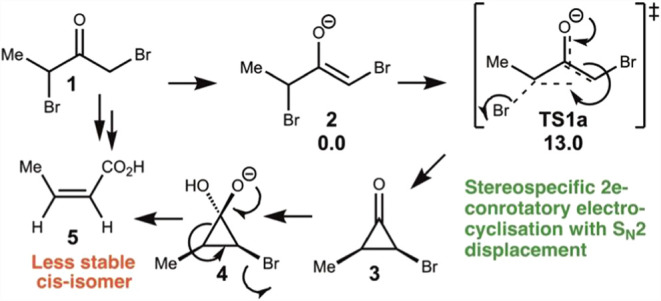

## Introduction

In 1963, Rappe published the first of a series of papers on his improved methods for the synthesis of *cis*-2-alkenoic acids from α,α′-dibromoketones (Scheme 1).^1^ This culminated in his preparatively useful *Organic Syntheses* procedure in 1973.^2^ The Rappe rearrangement has been used to prepare cis-acrylic acids for use in mechanistic studies,^3^ medicinal chemistry,^4,5^ natural product synthesis^6^ and structure proof,^7^ and polymer chemistry.^8^

**Scheme 1:**
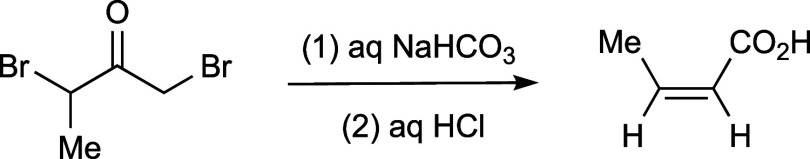
Rappe Rearrangement Producing (*Z*)/*cis*-2-Butenoic Acid (Isocrotonic Acid)

Rappe further investigated the general mechanism of this reaction. He was able to eliminate the α-ketocarbene and the semibenzilic acid rearrangement mechanisms and to provide ample support of the Favorskii mechanism as had been previously proposed by others.^9,10^

Although Rappe definitively addressed the general mechanism, he did not investigate what we consider to be the more remarkable aspect of this rearrangement: its extraordinarily high stereoselectivity for the less stable cis-isomers.^11^ That question had been raised by Zwanenburg in 1964, but he only commented that “The stereospecificity ... must involve concerted processes”.^10^ Because of our interest in using and extending the utility of the Rappe Rearrangement in organic synthesis, we sought a more detailed and satisfying explanation of the outcome.

In 1965, Woodward and Hoffmann published their analysis of the stereospecific disrotatory ring opening of cyclopropyl-X (X = halogen, tosylate, etc.) compounds to the allyl isomers (Scheme 2).^12^ Their analysis shed considerable light on the reaction, earlier described by Roberts^13^ and more extensively developed by DePuy^14,15^ and others. This led to the Woodward–Hoffmann–DePuy rule, which accounts for the torquoselectivity of the reaction: substituents R cis to the departing X-group rotate “in”. The DePuy rearrangement (Scheme 2) has been analyzed computationally by de Lera.^16,17^

**Scheme 2:**
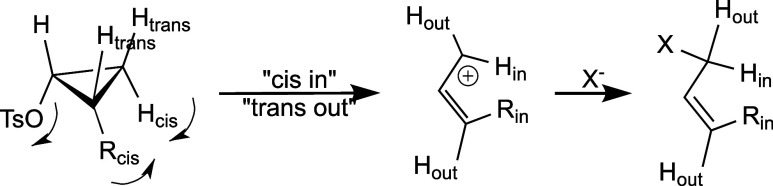
Stereochemical Outcome of the DePuy Rearrangement

While orbital symmetry,^12^ Favorskii,^1^ or torquoselectivity^14^ arguments may apply to the Rappe Rearrangement, it is by no means clear how without the aid of more experiments or computations or both. To begin, we broke down the Rappe problem by a stepwise reversal of what is known:

Step 2: The *cis*-alkenoic acid must arise from stereospecific ring opening of a bromocyclopropanone or its hydrated (ketal) form. Because the electronics are so different from those in the DePuy case, it is not yet obvious which factors will control the rotation of the alkyl substituent corresponding to the “*cis*-in” stereochemistry observed for the DePuy reaction.

Step 1: Once it is established which vicinal cyclopropanone isomer preferentially forms the *cis*-alkenoic acid product, we must then explain how and why that isomer is formed. If the geminal R, Br cyclopropanone is formed, it should produce the α-methyl acrylate and not the crotonate. There have been several suggestions regarding the mechanism of cyclopropanone formation from α-halo ketones. Sorensen has studied that step of the Favorskii Reaction computationally.^49^ Cyclopropanone might form via synchronous syn- or anti-elimination of H–Br from several possible orientations such as the W, syn-diaxial, or anti-diaxial conformations.^18–20^ Cyclopropanone itself might arise via an oxyallyl cation mechanism, which would require a determination of the stereochemical preferences of the oxyallyl reactant.^21,22^

Step 0: The reactive conformation must be accessible from the ground state conformation of the dihalo starting materials. The wide range of known Rappe precursors − acyclic, cyclic, with various substitution patterns − provide ample experimental evidence that reactive conformations are accessible regardless of small differences in the ground states which are not computed here.

## Results and Discussion

### Methodology

The details and results of the applicable computations are presented below. Four pathways in total are considered computationally, proceeding via TS1-TS5 and TS8 (Schemes 3 and 4). The energetics Δ*G*_298_ were obtained from a DFT model; ωB97XD^23^/Def2-TZVPP^24^/SCRF^25^­(CPCM) = continuum water using free energies obtained from calculation of the normal vibrational frequencies and the reactants and products deriving from following the intrinsic reaction coordinates (IRCs) obtained at the Def2-SVPP basis level to achieve reasonable computational cost for the larger systems, using the HPC integrator algorithm.^26^ Selected important transition states (TS1, TS2) were also evaluated at the computationally more costly Def2-TZVPP level with minimal differences observed. The Gaussian 16 program, Rev. C.01 and C.02 was used throughout.^27^ All data including input, output, and checkpoint files can be obtained from a FAIR data repository.^45^

**Scheme 3:**
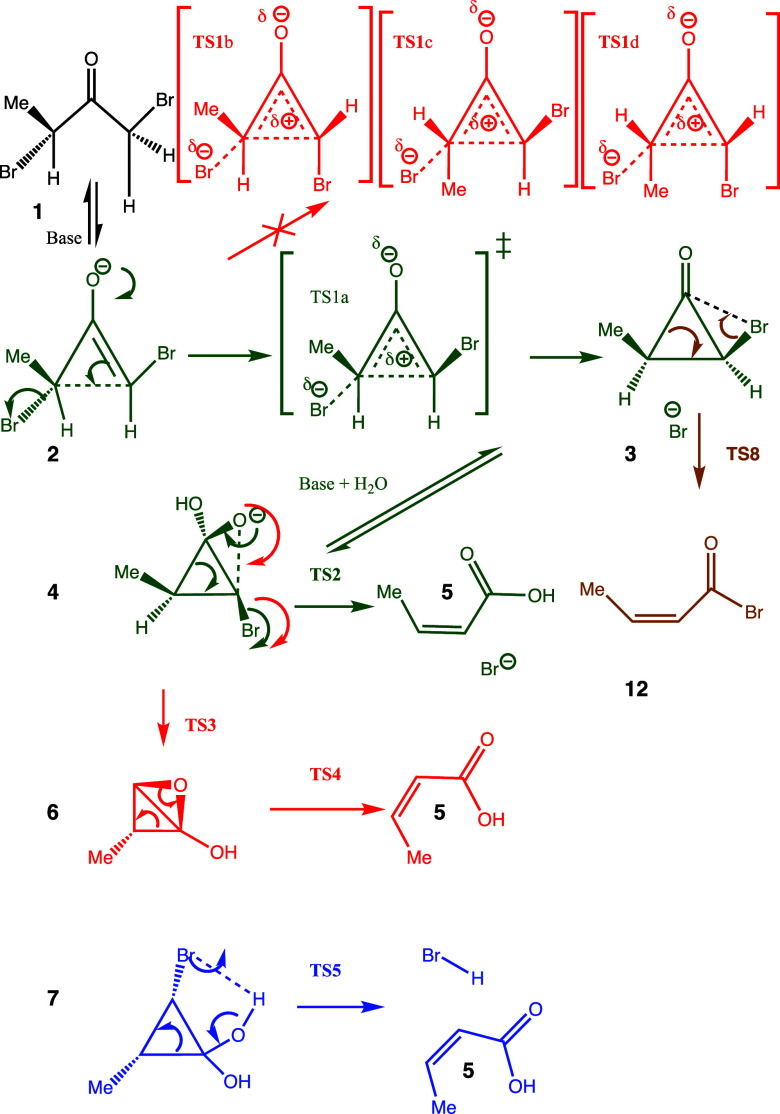
Mechanistic Possibilities for the Rappe Rearrangement; the Green Pathway Is the Most Energetically Favored

**Scheme 4:**
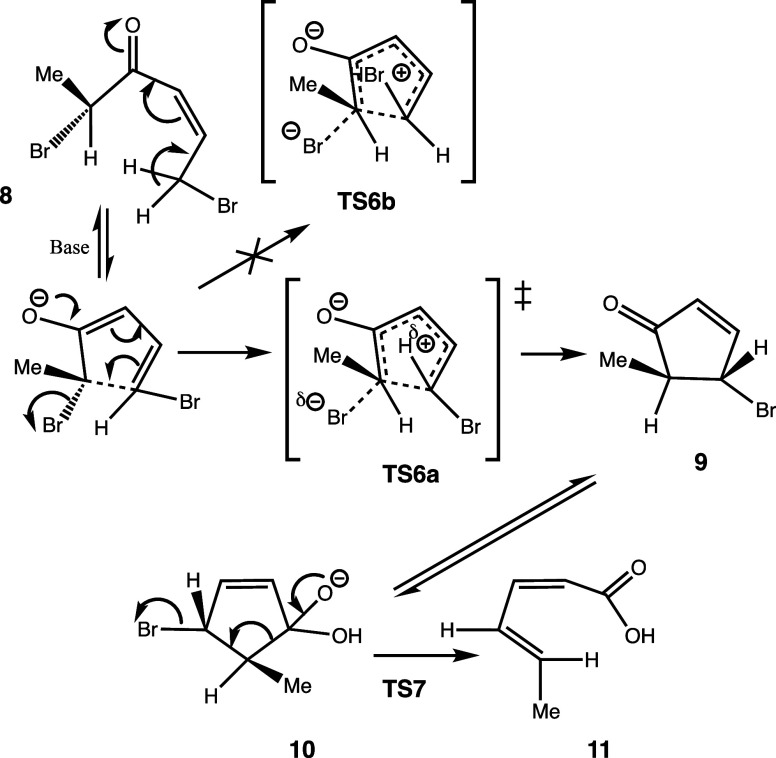
Mechanism for the Vinylogous Rappe Reaction

We first comment that Δ*G*_298_ for *cis*-2-butenoic acid is computed as 2.1 kcal/mol higher than the *trans*-isomer^30^ at the ωB97XD/Def2-TZVPP/SCRF = continuum water level, in agreement with the trans preference noted previously.^11^

#### TS1 (Scheme 3)^28^

The mechanism starts by forming the more stable (trisubstituted) enolate **2** of the α,α′-dibromoketone **1** by proton removal by the action of mild base (bicarbonate), using a methyl group as a stereochemical marker. This is followed by concomitant S_N_2-type cleavage of a C–Br bond oriented approximately anti to the plane of the nucleophilic enol anion π-system (Figure 1, stereoisomer TS1a). An alternative regioisomeric tetra-substituted enol pathway involving alternate proton removal proceeds via a transition state that is 6.3 kcal/mol higher in free energy than TS1a. The mechanistic type could be viewed as either a solvolytically assisted two-electron electrocyclization or an electrocyclically assisted solvolysis. Inversion of configuration at the carbon center associated with S_N_2-type displacement to form a nascent allylic cation occurs synchronously with the disrotatory electrocyclization of the allylic cation to form the three-membered cyclopropanone **3** (Scheme 3).

**Figure 1:**
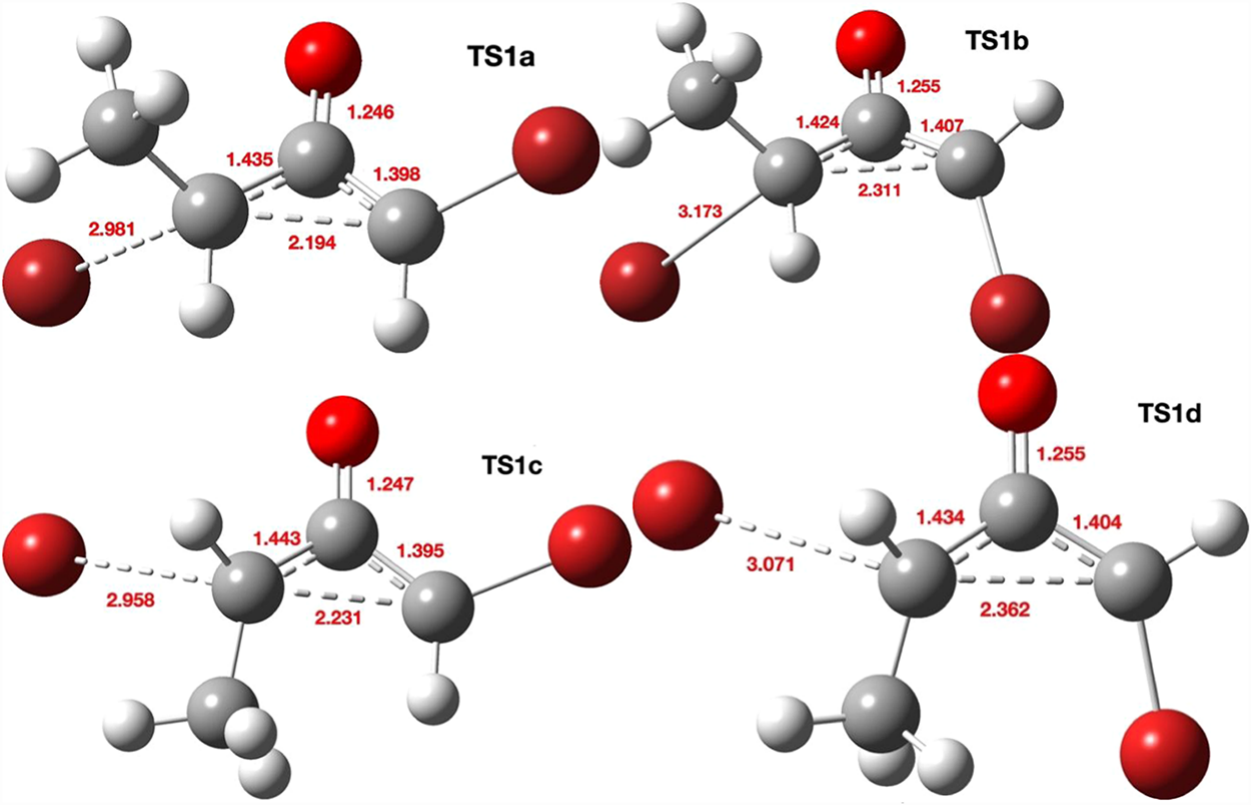
Four stereoisomeric forms for TS1a–d. Bond lengths are given in Å. The computed relative free energies (kcal/mol) at the Def2-TZVPP basis level are 0.0, 6.6, 5.2, and 13.8, respectively.

Three further stereoisomers TS1b, TS1c, and TS1d (Figure 1,b–d) are, respectively, 6.6, 5.5 kcal/mol and 13.8 higher in energy due to a combination of increased steric repulsions between the two inward-facing groups (H,H vs H,Br or Me,H or Me,Br, respectively) and a preference for *anti*- rather than *syn*-planar elimination, resulting in a clear-cut energetic preference for the stereoisomer TS1a (Figure 1). The geometries of the four stereoisomeric transition states are shown in Figure 1, with IRC pathways shown in Figure 2. Calculations predict a clear-cut stereochemical outcome for the formation of **3** as having the two ring hydrogens *cis* rather than *trans*.

**Figure 2:**
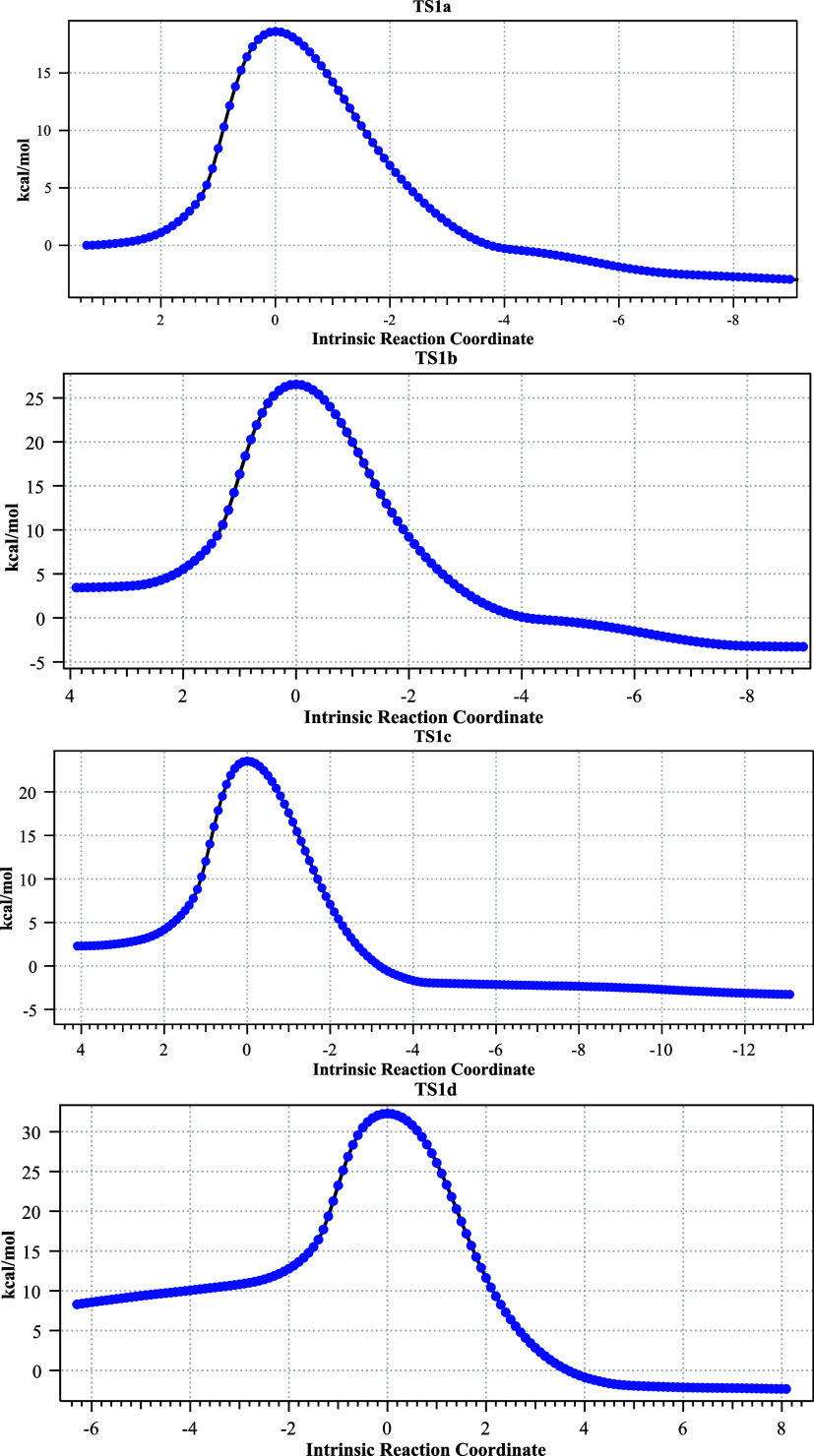
Computed IRC paths for the four stereoisomeric forms for TS1a–d at the Def2-SVP basis level, relative to the starting energy of **2**.

The free energy barrier (Def2-TZVPP) for the lowest energy stereoisomer TS1a (Figure 1) is a reasonable 13.0 kcal/mol (Table 1).

**Table 1:** Calculated Free Energies for Species **1**–**5**, **7** and Transition States **TS1a**–**TS5**, **TS8** at the ωB97XD/Def2-TZVPP/SCRF-CPCM = Water Level

species	total free energy^a^, Hartree	relative free energy, Δ*G*_298_, 1 atm., kcal/mol
**1**	–5531.51808	+24.4
**2**	–5531.55689	0.0
**TS1a**	–5531.53612	+13.0
**3**	–5531.56924	–7.8
**4**	–5531.61329	–35.4
**TS2**	–5531.60608	–30.9
**TS3**	–5531.57748	–12.9
**TS4**	–5531.58402	–17.0
**7**	–5531.57804	–13.3
**TS5**	–5531.54195	+9.4
**TS8**	–5531.53777	+12.0
**5**	–5531.73237	–100.1

a The total free energy is useful for data discovery using searches of the type shown below to reveal the molecular components of each energy: *e*.*g*., https://commons.datacite.org/doi.org?query=subjects.subjectScheme:Gibbs_Energy+AND+subjects.subject:\-5531.536*.

The model can be made more realistic by ensuring that **2** is solvated using, *e*.*g*., Na^+^·4H_2_O, retaining inclusion of the implicit continuum SCRF solvation model. The free energy barrier Def2-TZVPP to the reaction increases slightly to 14.7 kcal/mol, with the difference between TS1a and, *e*.*g*., TS1b being 7.6 kcal/mol; these values are not significantly altered from the uncoordinated model. The coordination by Na^+^·4H_2_O stabilizes the solvolytic phase via the departing bromide anion, which now clearly precedes the electrocyclization in a more S_N_1-like reaction via a two-step reaction (Figure 3), concluding with disrotatory electrocyclization (Figure 4).

**Figure 3:**
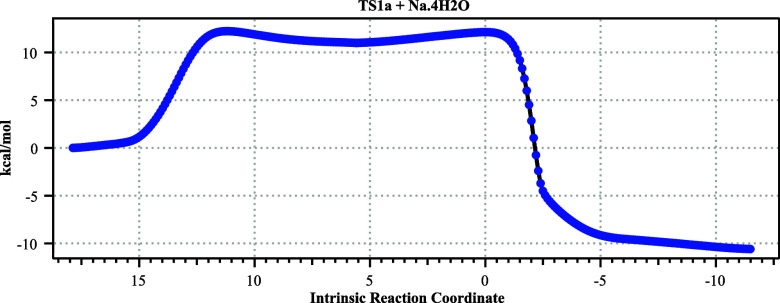
Computed IRC path TS1a coordinated with Na^+^·4H_2_O at the Def2-SVP basis level, showing a two-stage reaction involving a shallow minimum intermediate ion-pair (located at IRC = 6).

**Figure 4:**
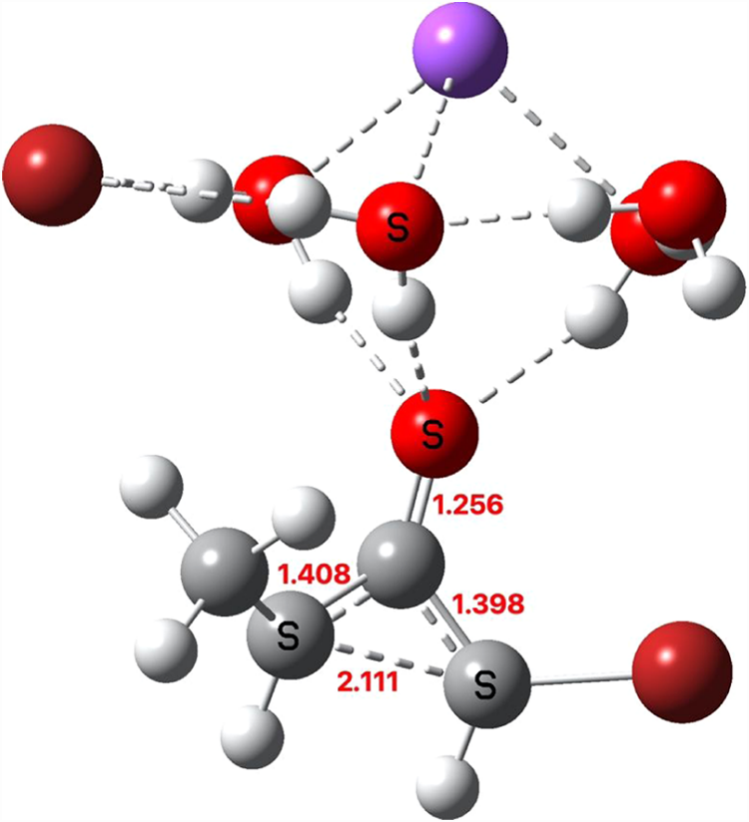
Geometry computed for TS1a·Na^+^·4H_2_O (Def2-TZVPP), bond lengths in Å.

#### TS2 ^29^

The next stage is to investigate whether this relative stereochemistry is maintained in reasonable subsequent mechanistic routes to the final *cis*-2-alkenoic acid product **5**. With this pathway, the cyclopropanone stereoisomer formed from TS1a to give **3** involves an exoenergic (−28.6 kcal/mol) hydration of the cyclopropanone to the cyclopropane diol, followed by further proton removal using base to form the cyclopropanediolate **4** and finally ring opening of **4** via TS2 to give the observed *cis*-2-alkenoic acid **5**. Similarly, the stereoisomer formed from higher energy TS1b also preserves the stereochemistry of **3** to give the *trans*-isomer of **5**. The calculated transition states for these two processes (Figures 5 and 6) show a discrimination of 1.7 kcal/mol in favor of *trans*-isomer ring opening of **4**, with a small free energy barrier of 2.8 and 4.5 kcal/mol for the corresponding *cis*-isomer.

**Figure 5:**
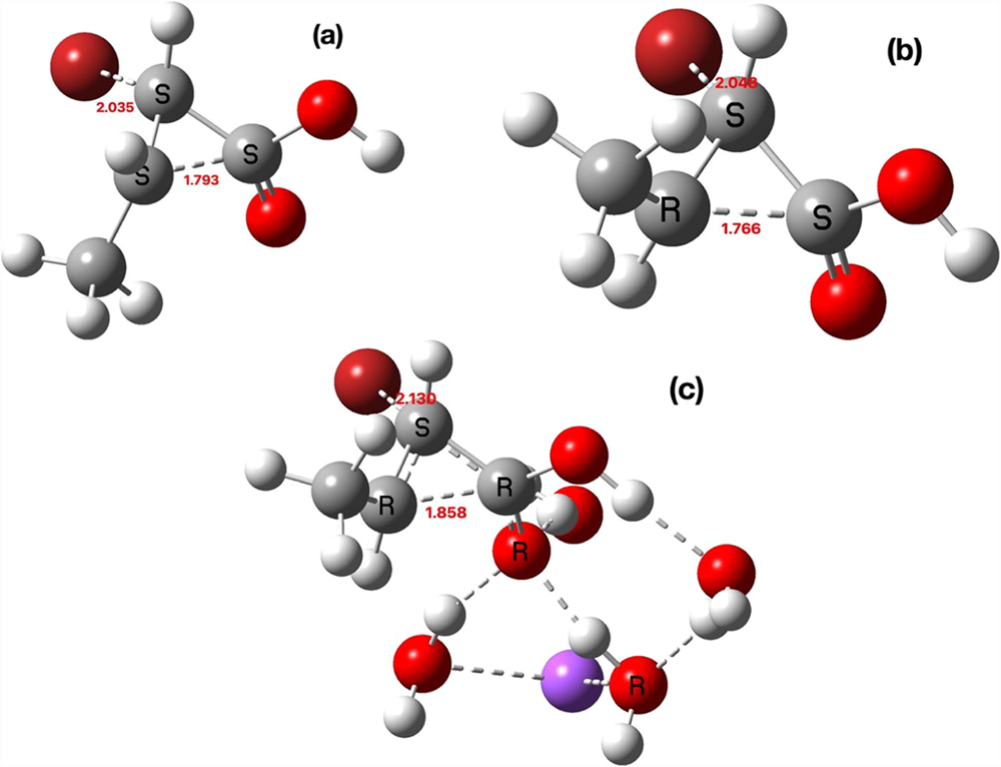
(a) TS2 for reactant **3** as formed from TS1a (b), TS2 for reactant **3** deriving from TS1b, and (c) TS2 for reactant **3** with Na^+^·4H_2_O as formed from TS1b, bond lengths in Å.

**Figure 6:**
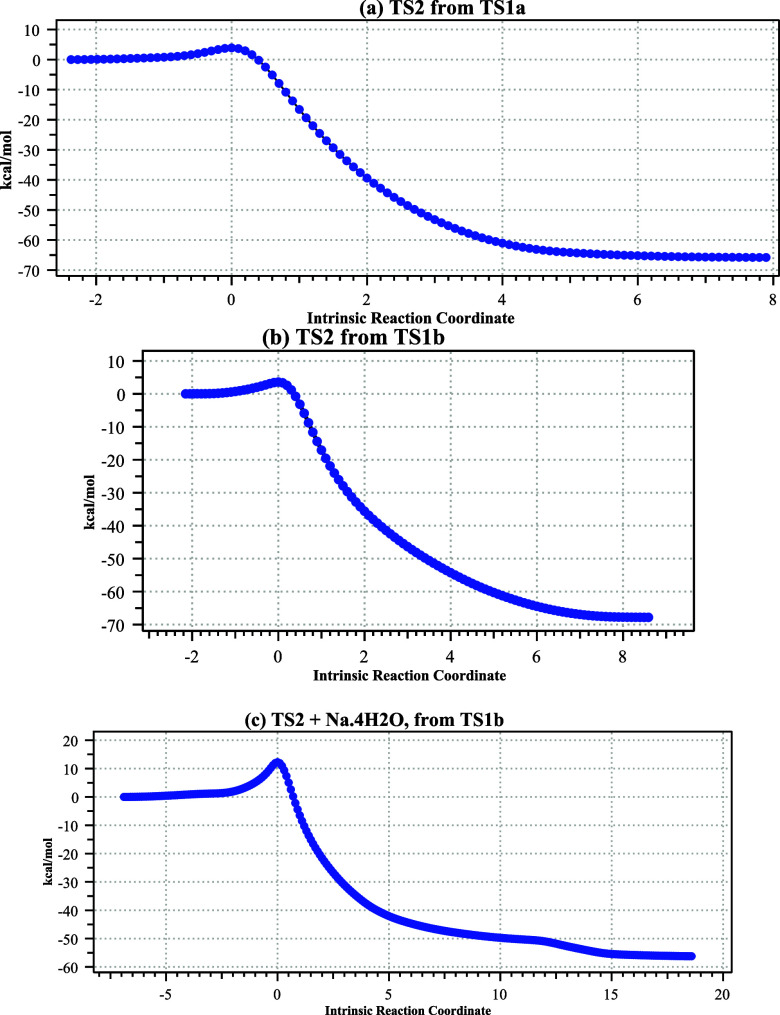
Computed IRC paths for TS2 for (a) **3** derived from TS1a, (b) **3** derived from TS1b, and (c) **3** derived from TS1b + Na·4H_2_O.

By this stage, however, the relative stereochemistry of the product is fixed and the trans preference of TS2 has no effect on the overall stereocontrol of the reaction, which is determined entirely by the preceding rate-determining step TS1a. Augmenting the model with Na^+^·4H_2_O increases the trans reaction free energy barrier to a more realistic 9.0 and to 11.1 kcal/mol for the corresponding cis isomer (Figures 5 and 6).

The overall computed energetics for the sequence **1** to **5** are shown in Table 1 ^30^ and Figure 7 for the aggregate atom assembly C_4_H_8_O_3_Br_2_, showing that the overall reaction is exoenergetic and that the rate-determining step is **TS1a** with respect to **2**. The conversion of **1** to **2** is a fast pre-equilibrium.

**Figure 7:**
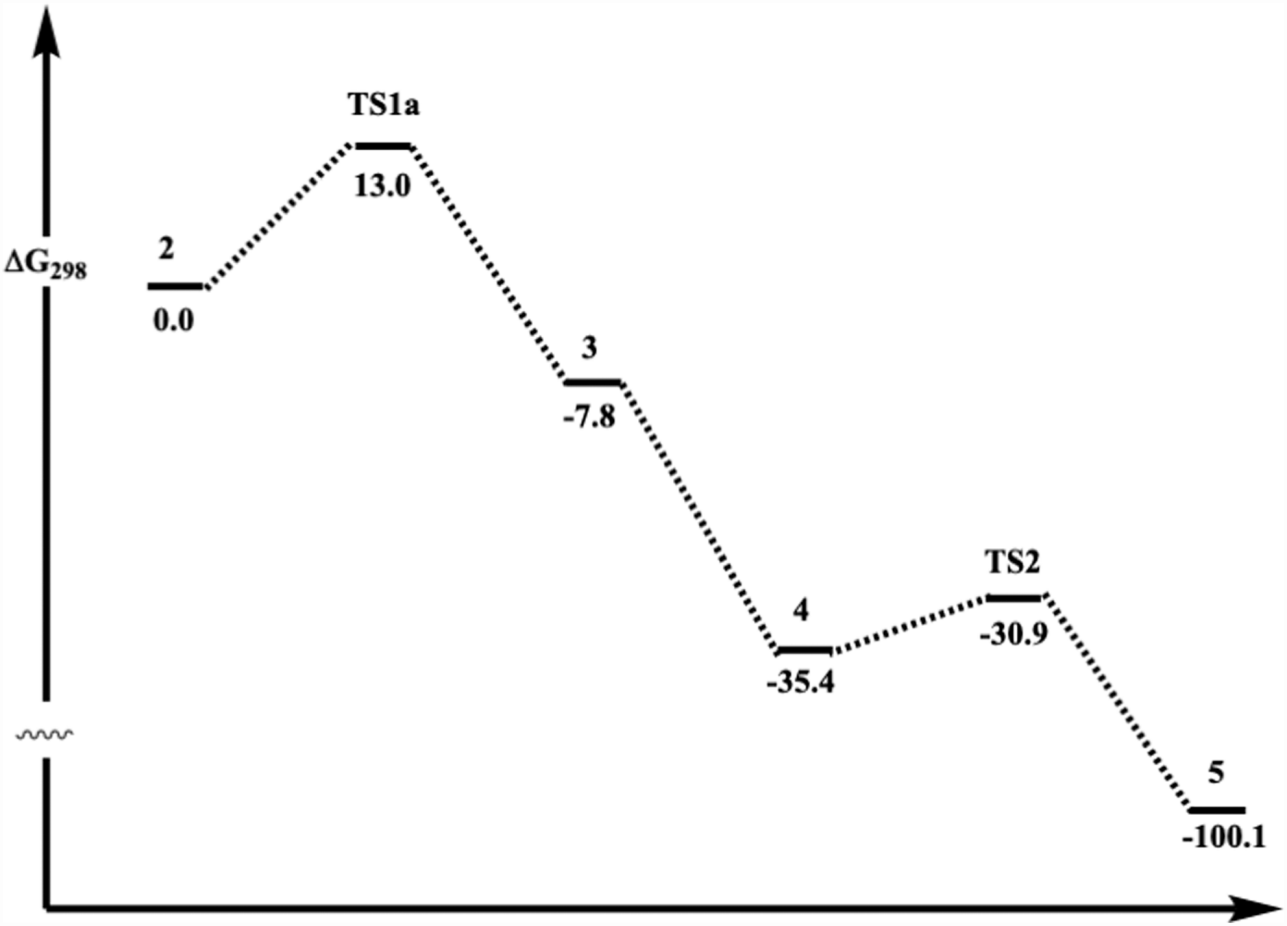
Schematic representation of the relative free energies (kcal/mol) for species involved in the lowest free energy pathway to the formation of **5**.

#### TS3 + TS4 ^31^

An alternative to TS2 is a two-step combination starting from **4** via TS3, in which the oxyanion of the diol carries out a direct S_N_2 displacement of bromide to form a bicyclic epoxide intermediate **6**, which then rearranges via TS4 (Scheme 3, red route) to the final cis-2-alkenoic acid **5** (Figure 8). From the same starting point as TS2, the free energy barrier for TS3 is 24.5 kcal/mol; using a Na^+^·4H_2_O model, it is 36.4 kcal/mol. These values are significantly higher than those for TS2 itself, suggesting that it is unlikely that the combined TS3 + TS4 pathway (Figure 9) will be followed in preference to the former.

**Figure 8:**
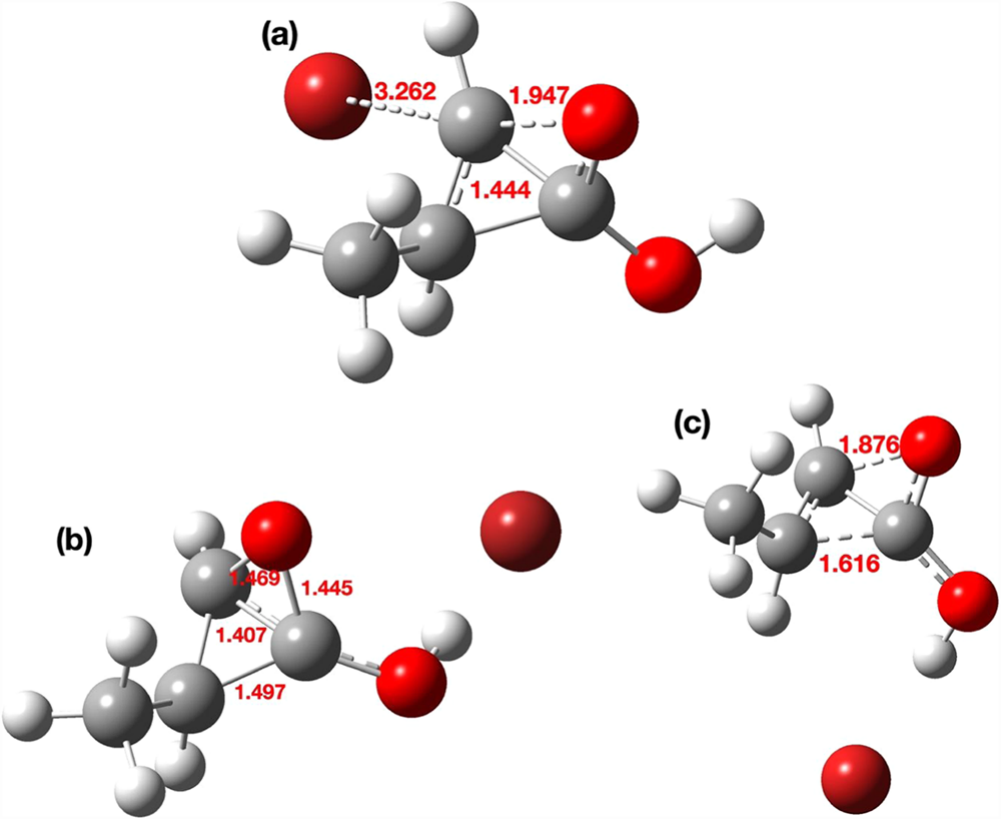
Geometries computed for (a) TS3, (b) intermediate **6**, and (c) TS4. All bond lengths are given in Å.

**Figure 9:**
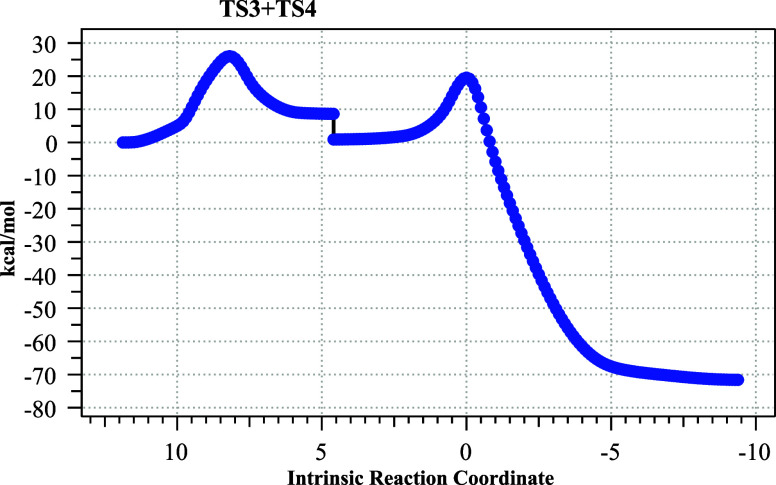
IRC for the combined pathways from TS3 and TS4. The discontinuity at IRC 4.5 is the result of OH···Br hydrogen bond formation for **6**, which is a process not included in the IRC pathways.

#### TS5 ^32^

The third alternative proceeds not from the oxyanion of the diol but from the neutral diol **7** via TS5 (Figures 10 and 11) and may be thought of as a [2 + 2 + 2] cycloreversion (Scheme 3, blue route). The relative free energy of TS5 from the diol **7** is 19.6 kcal/mol (*trans*) and 22.6 kcal/mol (*cis*), both higher than that for TS2 from the diolate **4**; the ring stereochemistry of the original cyclopropanone **3** is again retained by this reaction.

**Figure 10:**
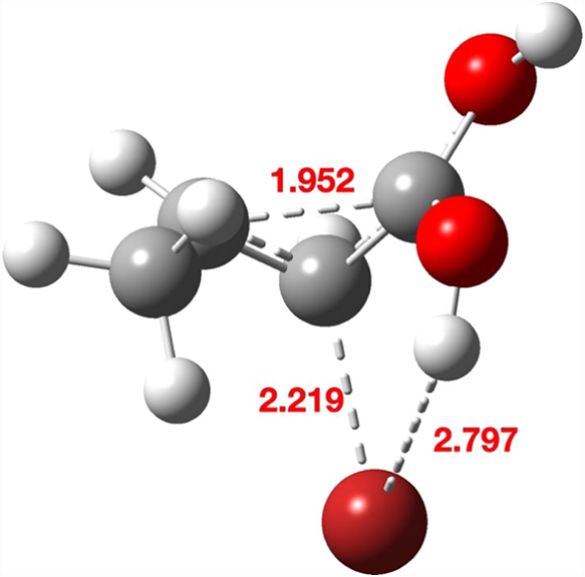
Geometry computed for TS5, with bond lengths in Å.

**Figure 11:**
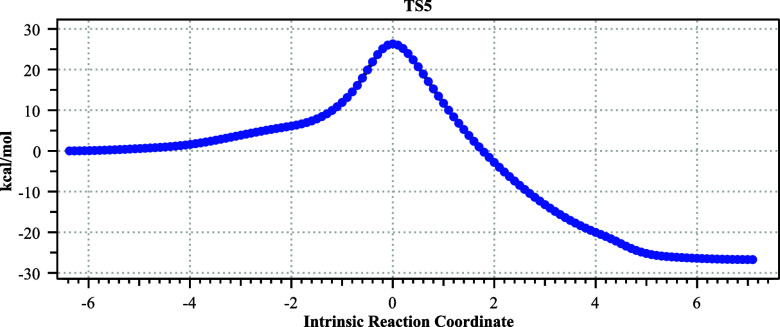
IRC for the pathway from TS5.

These computed pathways following Scheme 3 show that the stereochemical preference is developed at the first stage via TS1 (as TS1a) and is then retained in the alternative routes to the final 2-alkenoic acids, of which TS2 (green route) appears to be the favored path over TS3-TS5. This stereochemistry derives from disrotatory/suprafacial electrocyclization of a two-electron allyl cation-like species as induced by solvolysis of the bromide.

#### TS6 + TS7

The Vinylogous Rappe:^33^ The question then arises of whether the reaction can be extended to, *e*.*g*., **8**. Having an additional two electrons, this would now react via a nascent four-electron pentadienyl-like cation as shown in Scheme 4, with conrotatory/antarafacial four-electron electrocyclization replacing the disrotatory/suprafacial two-electron mode of **2** and proceeding instead via TS6a (Figure 12) to give **9**. The free energy barrier ΔG for TS6a is 13.8 kcal/mol, whereas the value for stereoisomer TS6b is 3.4 kcal/mol higher at 17.2 kcal/mol—induced at least in part by steric repulsions between the methyl group and the bromine. This illustrates how two-electron homologation (as predicted by the Woodward–Hoffmann rules) changes the stereochemical outcome for species **9**. Overall, these reaction barriers are very similar to the reaction of lower homolog **1**. Species **9** then hydrates and deprotonates to give **10**, which rearranges to the trans alkene **11** via **TS7** (Figure 13), overcoming a free energy barrier from **10** of 10.1 kcal/mol.

**Figure 12:**
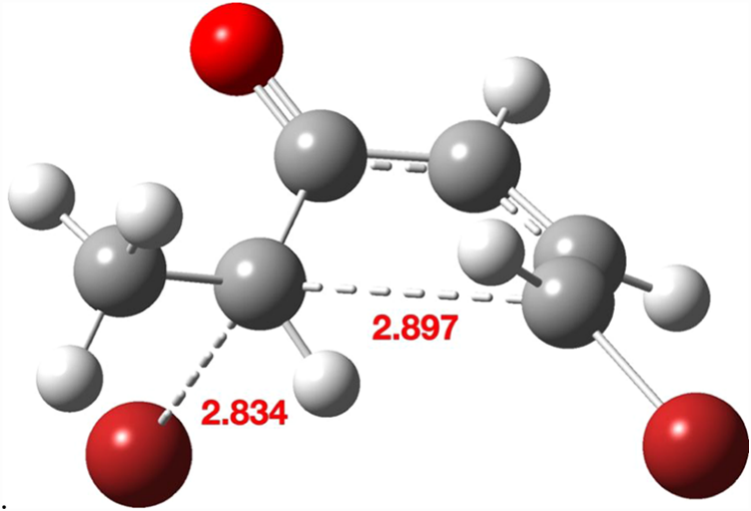
Conrotatory cyclization via TS6a showing antarafacial C–C bond formation. Bond lengths in Å.

**Figure 13:**
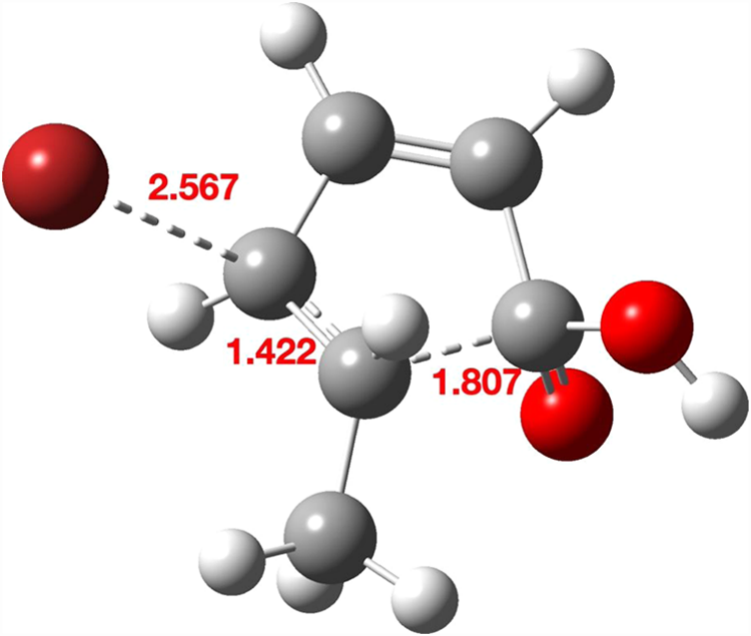
Geometry computed for TS7, bond lengths in Å.

#### TS8 (Scheme 3)^34^

The reactions described above are conducted in aqueous solutions, and water actively participated in the mechanism. We have also investigated what might happen to the cyclopropanone intermediate **3** in a nonaqueous medium. Thus, enantiopure *trans*-3,5-di-*tert*-butylcyclopropanone is known to racemize at higher temperatures in such solvents^35^ and cyclopropanones are also known to undergo 2 + 4 cycloaddition reactions with dienes such as furan.^36^ Thus, species **3** can be envisaged as undergoing a ring opening to give an acyl bromide directly (Scheme 3), which would then be hydrolyzed to the butanoic acid by addition of water. The transition state **TS8** for such a process (Figure 14) has a free energy barrier of 20.0 kcal/mol for the *trans*-isomer and 21.8 kcal/mol for the *cis*-isomer, both of which have relatively low thermal barriers. As before, the stereochemistry of the starting cyclopropanone determines the configuration of the final product.

**Figure 14:**
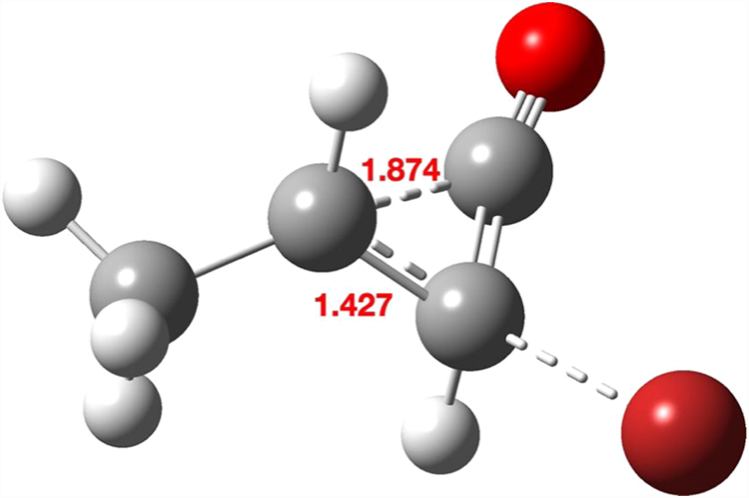
Geometry computed for TS8, with bond lengths in Å.

The IRC profile (Figure 15) shows that the concerted reaction proceeds in two stages. The first stage (IRC 2 to −2) culminates in the formation of an oxenium-like cation paired with a bromide anion (a “hidden intermediate”, visible at IRC ∼ −3 in the gradient norm plot) followed by attack of the free bromide on the oxenium cation to form the acyl bromide. The ionic nature of this process is illustrated by the evolution of the dipole moment along the IRC.

**Figure 15:**
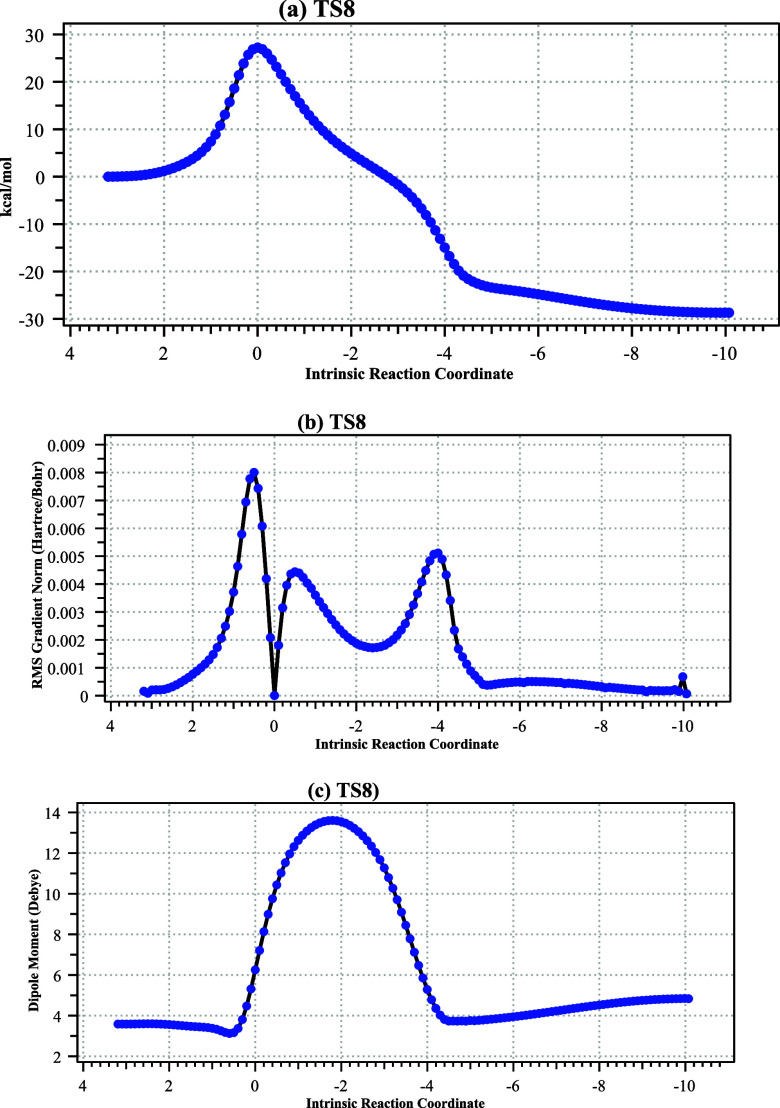
IRC for pathway from TS5 showing (a) relative energy, (b) gradient norm, revealing a hidden intermediate at IRC = −3, and (c) the dipole moment response.

We predict therefore that the initial cyclopropanone product **3** bearing a bromide leaving group, as formed in the standard Rappe reaction, could also be used to stereoselectively produce butenoic acid **5** in nonaqueous media. One potential example of this reaction has been reported.^37^ Furthermore, there are examples of amide formation under nonaqueous conditions when α,α′-dihaloketones are treated with amine bases.^38–40^

## Conclusions

The mechanistic scenarios set out in Scheme 3 for the base-catalyzed rearrangement of α,α′-dibromoketones **1** to *cis*-2-alkenoic acids can be quantified using DFT calculations. This identifies a stereospecific first step for the ring closure to cyclopropanone **3**, which follows the Woodward–Hoffmann rules for a two-electron allylic cation, itself induced by departure of the first bromine atom. Although this mechanism favors an S_N_2-type mechanism when modeled without explicit solvating water and sodium cation, it morphs into an S_N_1 type with a shallow ion-pair intermediate when these components are added to the model. The stereochemical preference for disrotatory/suprafacial bond formation is, however, retained in both mechanistic extremes. Of the three alternatives for hydration of the cyclopropanone and then elimination of HBr, the direct migration pathway via TS2 appears favored, but all three paths in fact proceed with retention of the stereochemical preference induced by the first step. This then forms a satisfactory explanation of the remarkable cis-preference identified by Rappe all those years ago.

Armed now with the orbital symmetry explanation for this selectivity, one can speculate upon new variations of the Rappe Rearrangement. An example of a homo-Rappe Rearrangement was presented above (Scheme 4): the homologated mechanism in which an extra two electrons are added via an additional alkene bond should proceed to form a *trans*-4-hexa-dienoic acid.

Hetero-Rappe analogues (Scheme 5) may also provide convenient routes to new compounds. The episulfoxide case (Scheme 5, X = S) and episulfone case (X = SO) might be compared to the Weinges^41^ and Ramberg-Backlund Reactions,^42^ respectively. In particular, an Aza-Rappe (X = N) might be used to prepare otherwise difficult-to-obtain *cis*-nitroalkenes for Diels–Alder or polymerization reactions. Some of these possibilities for a generalized Rappe rearrangement are currently being investigated.^43^

**Scheme 5:**
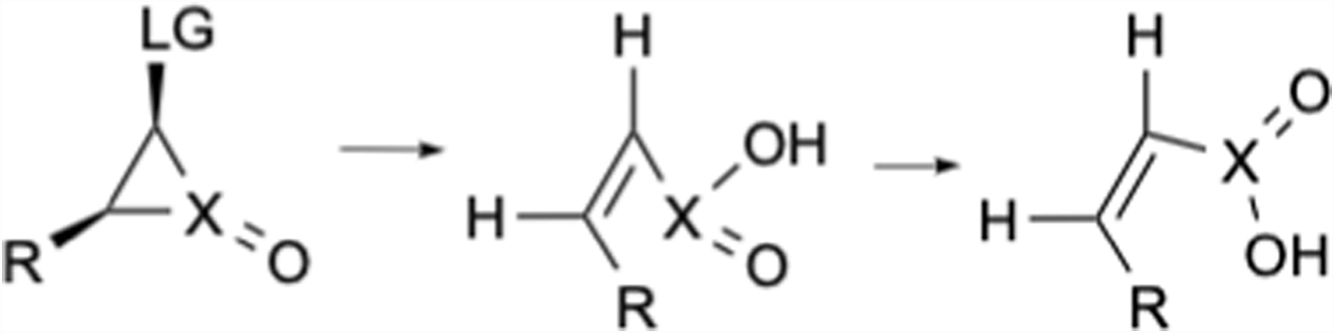
Generalized Hetero-Rappe Rearrangement

## Supplementary Material



## Data Availability

The data underlying this study are available in the published article, in its Supporting Information, and openly available in the Imperial College Research Data Repository^44^ at DOI: 10.14469/hpc/11172,^45^ with data discovery^46^ enabled by searches of the DataCite repository metadata store.^47^

## Notes

Computed coordinates for key species, with each set provided with a link to the more complete repository-based FAIR data version (XYZ)
